# miR-186 regulates epithelial–mesenchymal transformation to promote nasopharyngeal carcinoma metastasis by targeting ZEB1^☆^

**DOI:** 10.1016/j.bjorl.2023.101358

**Published:** 2023-11-06

**Authors:** Liangke Tang, Yalang Xiang, Jing Zhou, Tao Li, Tingting Jia, Guobo Du

**Affiliations:** a Affiliated Hospital of North Sichuan Medical College, Department of Oncology, Nanchong, China; b North Sichuan Medical College, Nanchong, China; c Affiliated Hospital of North Sichuan Medical College, Department of Neurology, Nanchong, China; d Department of Oncology, People’s Hospital of Nanbu County, Nanchong, China; e The First Affiliated Hospital of Jinan University, Tianhe, China

**Keywords:** miR-186, ZEB1, Nasopharyngeal carcinoma, Epithelial–mesenchymal transformation, Metastasis

## Abstract

**Objectives:**

Nasopharyngeal carcinoma (NPC) is an aggressive epithelial cancer. The expression of miR-186 is decreased in a variety of malignancies and can promote the invasion and metastasis of cancer cells. This study aimed to explore the role and possible mechanism of miR-186 in the metastasis and epithelial–mesenchymal transformation (EMT) of NPC.

**Methods:**

The expression of miR-186 in NPC tissues and cells was detected by RT-PCR. Then, miR-186 mimic was used to transfect NPC cell lines C666-1 and CNE-2, and cell activity, invasion and migration were detected by CCK8, transwell and scratch assay, respectively. The expression of EMT-related proteins was analyzed by western blotting analysis. The binding relationship between miR-186 and target gene Zinc Finger E-Box Binding Homeobox 1 (ZEB1) was confirmed by double luciferase assay.

**Results:**

The expression of miR-186 in NPC was significantly decreased, and transfection of miR-186 mimic could significantly inhibit the cell activity, invasion, and migration, and regulate the protein expressions of E-cadherin, N-cadherin and vimentin in C666-1 and CNE-2 cells. Further experiments confirmed that miR-186 could directly target ZEB1 and negatively regulate its expression. In addition, ZEB1 has been confirmed to be highly expressed in NPC, and inhibition of ZEB1 could inhibit the activity, invasion, metastasis and EMT of NPC cells. And co-transfection of miR-186 mimic and si-ZEB1 could further inhibit the proliferation and metastasis of NPC.

**Conclusion:**

miR-186 may inhibit the proliferation, metastasis and EMT of NPC by targeting ZEB1, and the miR-186/ZEB1 axis plays an important role in NPC.

## Highlights

• MiR-186 was lowly expressed in nasopharyngeal carcinoma.
• MiR-186 inhibited epithelial–mesenchymal transformation of nasopharyngeal carcinoma.
• MiR-186 directly targets ZEB1 to negatively regulate its expression.
• MiR-186 regulated the epithelial–mesenchymal transformation by targeting ZEB1.

## Introduction

Nasopharyngeal carcinoma (NPC) is a malignant cancer occurring in the epithelium of the nasopharynx mucosa and minor salivary glands.^1^ According to statistics, there were 133,354 new cases of NPC worldwide in 2020, and 80,008 deaths.^2^ More than 40% of NPC are concentrated in China, with higher incidence in the south of China.^3^ Epidemiological studies indicate that Epstein–Barr virus infection, genetic susceptibility, exposure to harmful environmental factors and special diet may be associated with its onset.^4^ Currently, the best treatment for NPC is a combination therapy based on radiotherapy, but multiple treatments have not significantly improved patient outcomes.^5^ Although the 5-year overall survival rate of NPC can reach about 80% after standardized treatment, there are still 10%‒15% of primary NPC patients with local or regional recurrence.^6^ NPC is the cancer with the highest metastasis potential among all head and neck cancers, and distant metastasis and local compound are the main causes for its treatment failure.^7^ Therefore, it is necessary to elucidate markers and molecular mechanisms independently associated with cancer progression and aggressiveness in order to provide patients with more effective treatment.

MicroRNA (miRNA), a class of single-stranded non-coding RNA composed of 22 nucleotides, inhibits the transcription and accelerates the degradation of target mRNA at the pre-transcription level by binding with 3′-UTR, thus affecting the intracellular transcript level.^8^ MiRNA presents different expression characteristics in different tissues, and there is increasing evidence that miRNA plays a key role in many diseases, including the progression of cancers.^9^ Understanding the molecular mechanism of cancer from the perspective of miRNA and mRNA regulation is helpful to provide clues for the molecular diagnosis and treatment of cancer.^10^ MiR-186 is a regulatory factor associated with the progression of various cancers.^11^ Multiple studies have shown that miR-186 is down-regulated in malignant cancers including cervical cancer,^12^ renal cell cancer,^13^ liver cancer^14^ and lung adenocarcinoma,^15^ and can be used as a cancer inhibitor. In a recent study, miR-186 was also found to be significantly under-expressed in NPC,^16^ suggesting that miR-186 may play a key role in the occurrence and progression of NPC. In addition, miR-186 is reported to be involved in the regulation of key cellular processes such as cell proliferation, apoptosis, and metastasis, all of which are important in the development of cancer. However, whether the expression of miR-186 in NPC is related to malignant metastasis of NPC still need further study.

The purpose of this study was to explore the expression of miR-186 in NPC and its effects on the proliferation and metastasis of NPC. Based on bioinformatics analysis, the downstream targets of miR-186 were identified, and the role and possible mechanism of miR-186 in NPC were explored using the miRNA–mRNA regulatory network, so as to further understand the molecular mechanism of the progression and metastasis of NPC.

## Methods

### Tissue sample collection

Thirty pairs of NPC and para-cancer tissue samples were obtained from NPC patients in the Affiliated Hospital of North Sichuan Medical College. All patients signed informed consent of specimen retention before surgery, and all patients conforming to surgical conditions routinely collected pathological tissue samples. Professional doctors ensure that the NPC sample and its adjacent normal tissue are removed as accurately as possible during the procedure. Detailed pathological analysis was performed on the collected tissues, such as immunohistochemical staining to detect the expression of specific tumor markers in neighboring tissues, and cell types in neighboring tissues were further confirmed through experiments such as cell morphology observation. All specimens were confirmed by 2 or more pathologists in our hospital as NPC tissues and adjacent normal tissues, and no tumor cells and atypical hyperplasia cells were found in normal tissues. This study was approved by the Medical Ethics Committee of Affiliated Hospital of North Sichuan Medical College (No. 2022ER287-1).

### Cell culture

Human nasal epithelial cell line HNEpC (nº BFN60806616) and human NPC cell line C666-1 (nº BFN608006727) and CNE-2 (nº BFN60700251) were purchased from BLUEFBIO (Shanghai, China). The three cells were cultured in DMEM high glucose medium (Hyclone, USA) containing 10% fetal bovine serum (FBS, Gibco, USA) and 1% penicillin-streptomycin solution (Hyclone, USA) in a 5% CO_2_ incubator at 37 °C.

### Cell transfection

The NC mimic (nº miR1N0000001-1-5) and the hsa-miR-186-5p mimic (nº miR10000456-1-5) were purchased from Ribobio (China). Interference sequences of si-RNA target Zinc Finger E-Box Binding Homeobox 1 (ZEB1) were also synthesized by Ribobio (China). Then, NC mimic, miR-186 mimic, si-NC and si-ZEB1 were used to transfect C666-1 and CNE-2 cells by Lipofectamine 2000 (nº 11668019; Invitrogen, USA). First, mimic, siRNA and lipofectamine 2000 reagents were diluted in Opti-MEM, incubated for 5 min, and the diluted reagents and plasmids were mixed and placed at room temperature for 20 min. When the cell density reached 70%, the transfection mixture was added and cultured at 37 °C with 5% CO_2_. After transfection for 6 h, fresh medium was exchanged, and the follow-up study was conducted 48 h later.

### Cell viability analysis

Cell viability was analyzed by Cell Counting kit-8 (CCK8). After different transfections, CCK8 solution (nº C0037; Beyotime, China) was added and incubated with cells at 37 °C for 1 h. Absorbance was measured at 570 nm using an enzyme marker, and the cell survival rate of each treatment group was calculated.

### Cell invasion assay

Cell invasion was carried out through transwell compartments coated with an artificial substrate gel Matrigel. The complete culture medium containing FBS was added to the lower chamber of the transwell, and 5 × 10^4^ single-cell suspension suspended with the serum-free medium was added to the upper chamber. The cells were cultured in a cell incubator at 37 °C for 24 h, and the number of invaded cells was observed after fixing with 4% paraformaldehyde solution (nº P0099; Beyotime, China) for 20 min, and staining with crystal violet staining solution (nº C0121; Beyotime, China) for 5–10 min.

### Cell migration assay

Cell migration was performed by a scratch assay. About 5 × 10^5^ cells were evenly spread in the six-well plate, and two parallel lines were drawn after sticking to the wall and growing. The cells were continued to be cultured in a 37 °C, 5% CO_2_ incubator for 24 h. Observed and photographed under a microscope, the migration distance was calculated.

### Double luciferase reporter gene assay

First, the Wild-Type (WT) and Mutant (MUT) sequences of ZEB-1 were constructed on luciferase vectors pGL3-basic. Luciferase vectors and miR-186 mimic or NC mimic were transfected into 293T cells according to experimental groups using transfection reagents Lipofectamine 2000. The dual luciferase reporter gene assay kit (nº RG027; Beyotime, China) was used to detect fluorescence intensity and analyze luciferase activity.

### Western blot analysis

The total protein in NPC tissue or cells was obtained by RIPA lysis buffer (nº P0013B, Beyotime, China), the protein content was quantified by BCA protein assay kit (nº P0012S, Beyotime, China), and the total protein was separated by SDS-PAGE after protein denaturation. Then, the isolated proteins were transferred to the PVDF membrane, sealed with 5% skim milk, and incubated at 4 °C overnight with the primary antibody β-actin (nº sc-8432; Santa Cruz Biotechnology, USA), ZEB1 (nº sc-515797; Santa Cruz Biotechnology, USA), E-cadherin (nº sc-8426; Santa Cruz Biotechnology, USA), N-cadherin (nº sc-8424; Santa Cruz Biotechnology, USA) or vimentin (no. sc-6260; Santa Cruz Biotechnology, USA). Then, the secondary antibody goat anti-mouse IgG-HRP (no. sc-2005; Santa Cruz Biotechnology, USA) was incubated at room temperature for 2 h. ECL western blotting substrate (no. PE0010; Solarbio, China) and gel imaging analyzer (Bia-Rad, USA) was used to visualize the images. The relative protein expression was calculated using β-actin as an internal reference.

### Real-time quantitative polymerase chain reaction (RT-PCR)

The gene expressions of miR-186 and ZEB1 in NPC tissue and cells were analyzed by RT-PCR. Total RNA was extracted by TRIzol reagent (nº 15596026; Invitrogen, USA). For the examination of miR-186, cDNA was obtained by miR-Quant TaqMan microRNA cDNA synthesis kit (no. MT0006; Biorab, China), and PCR reaction was performed by miR-Quant TaqMan microRNA qPCR kit (nº MTT02318; Biorab, Beijing, China). For the detection of ZEB1, cDNA was obtained by HiScript 1st strand cDNA synthesis kit (nº R111-01; Vazyme, Nanjing, China), and PCR reaction was performed by HiScript II one step RT-PCR kit (nº P611-01; Vazyme, Nanjing, China). The miR-186 and U6 sequence was provided by the kit, the ZEB1 sequence was F: 5′-ATGCAGCTGACTGTGAAGGT-3′, R: 5′-GCCCTTCCTTTCCTGTGTCA-3′, and the β-actin sequence was F: 5′-GAAGATCAAGATCATTGCTCC-3′, R: 5′-TACTCCTGCTTGCTGATCCA-3′. Using U6 as the internal reference of miR-186 and β-actin as the internal reference of ZEB1, the relative gene expression was calculated by the 2^−△△CT^ method.

### Statistical analysis

All data in this study were analyzed by SPSS software and expressed as mean ± standard deviation. The difference analysis between the two groups was conducted by the Student *t*-test, and the difference analysis among the multiple groups was conducted by one-way analysis of variance; *p* < 0.05 was considered statistically significant.

## Results

### MiR-186 was lowly expressed in NPC tissues and cells

**Figure 1:**
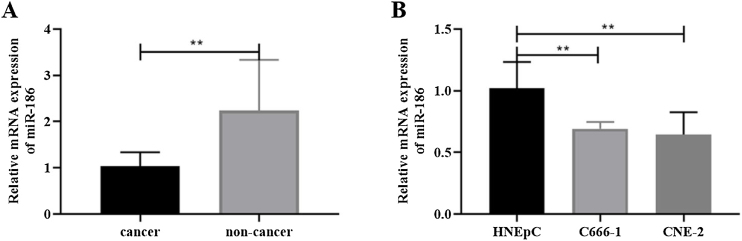
Expression of miR-186 in NPC. The expression of miR-186 in NPC tissues (A) and cells (B) was detected by RT-PCR. *p < 0 .05, **p < 0.01, ***p < 0.001.

To analyze the effects of miR-186 on NPC, the expression of miR-186 was analyzed by RT-PCR, and the results showed that the expression of miR-186 in cancer tissues was significantly reduced (*p* < 0.01, Fig. 1A). Similarly, miR-186 expression was significantly decreased in NPC cell lines C666-1 and CNE-2 compared to human nasal epithelial cell line HNEpC (*p* < 0.01, Fig. 1B).

### MiR-186 inhibited proliferation, metastasis, and EMT of NPC cells

**Figure 2:**
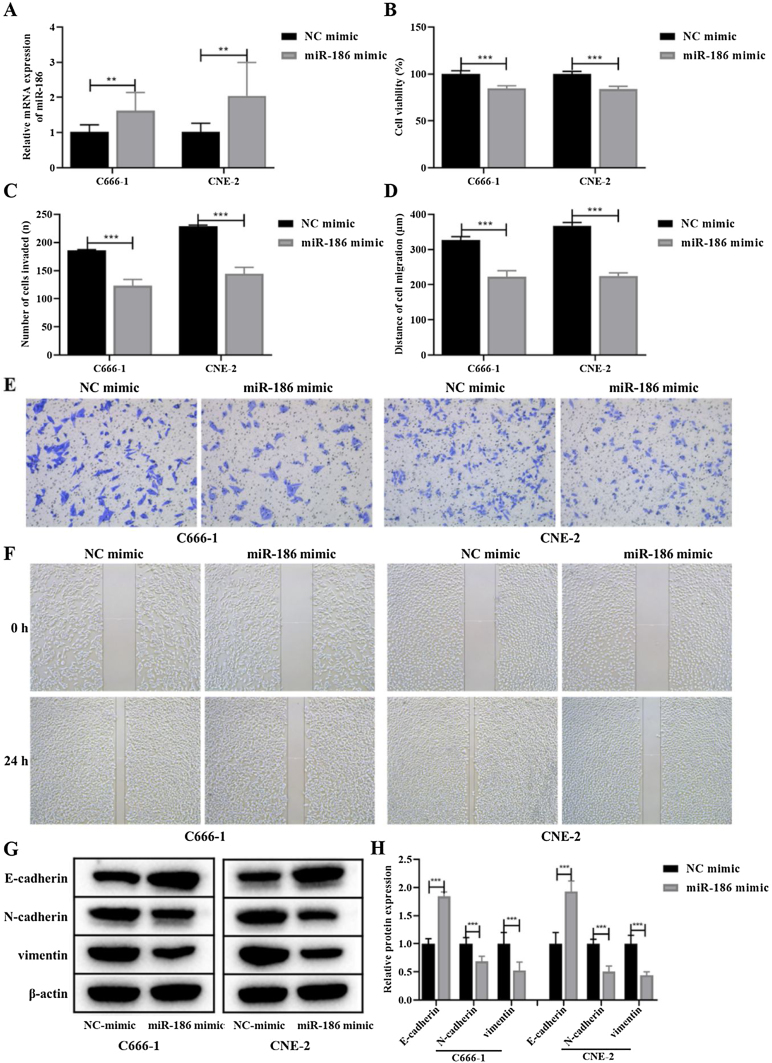
Effects of miR-186 on proliferation and metastasis of NPC cells. (A) The expression of miR-186 in cells transfected with miR-186 mimic was detected by RT-PCR. (B) Cell viability was detected by CCK8. (C) The number of cells invaded. (D) Cell migration distance. (E) Representative images of cell invasion measured by transwell. (F) Representative images of cell migration detected by scratch assay. (G) The protein expressions of E-cadherin, N-cadherin and vimentin were detected by WB. Full-length blots/gels are presented in Supplementary Fig. 1. (H) Gray scale analysis of protein bands. *p < 0.05, **p < 0.01, ***p < 0.001, vs. NC mimic.

Due to the low expression of miR-186 in C666-1 and CNE-2 cells, the miR-186 mimic was used to transfect C666-1 and CNE-2 cells, and RT-PCR was used to verify the expression of miR-186 after transfection. The results showed that the miR-186 mimic significantly increased the expression of miR-186 in two NPC cells compared with the NC mimic (*p* < 0.01, Fig. 2A). The effects of miR-186 mimic on cell proliferation and metastasis were further analyzed, CCK8 experiment showed that the cell viability in miR-186 mimic transfected cells was significantly reduced compared with NC mimic in both two NPC cells (*p* < 0.01, Fig. 2B). In addition, the invasion number (Fig. 2C and E) and migration distance (Fig. 2D and F) of C666-1 and CNE-2 cells in the miR-186 mimic group were significantly reduced compared with the NC mimic group (*p* < 0.001), suggesting that miR-186 mimic inhibited NPC metastasis. The expression of EMT-related proteins was further analyzed, and results showed that miR-186 mimic significantly promoted the protein expression of E-cadherin, reduced the protein expression of N-cadherin and vimentin, and inhibited the EMT of NPC cells compared with the NC group (*p* < 0.01, Fig. 2G and H).

### MiR-186 directly targets ZEB1 to negatively regulate its expression

**Figure 3:**
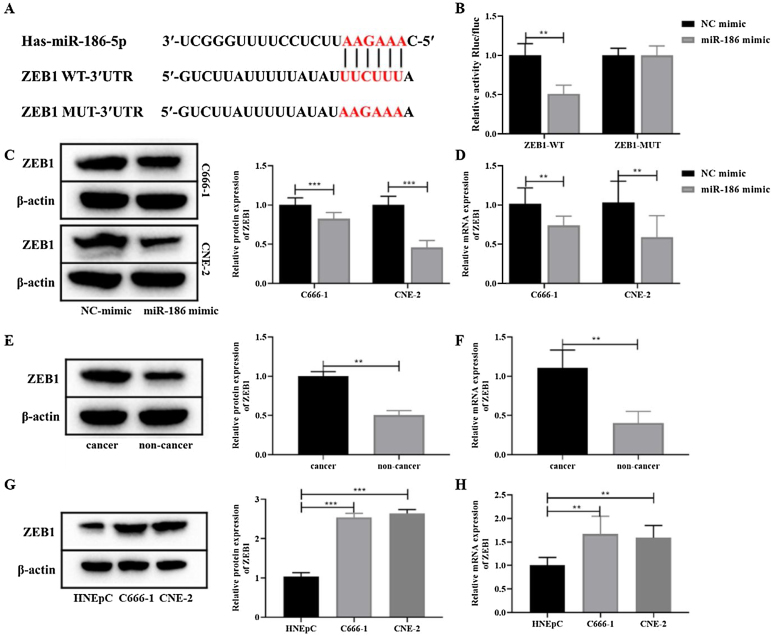
MiR-186 negatively regulated the expression of ZEB1 in NPC. (A) The predicted potential binding site between miR-186 and ZEB1. (B) Double luciferase reporter assay was used to detect luciferase activity. (C) The protein expression of ZEB1 in cells transfected with miR-186 mimic was detected by WB. Full-length blots/gels are presented in Supplementary Fig. 2. (D) The gene expression of ZEB1 in cells transfected with miR-186 mimic was detected by RT-PCR. (E) The protein expression of ZEB1 in NPC tissues was detected by WB. Full-length blots/gels are presented in Supplementary Fig. 3. (F) The gene expression of ZEB1 in NPC tissues was detected by RT-PCR. (G) The protein expression of ZEB1 in NPC cells was detected by WB. Full-length blots/gels are presented in Supplementary Fig. 4. (H) The gene expression of ZEB1 in NPC cells was detected by RT-PCR. *p < 0.05, **p < 0.01, ***p < 0.001.

To further clarify the mechanism of miR-186 regulating NPC metastasis, the potential downstream genes of miR-186 were predicted through the RNAInter bioinformatics platform. The results showed that miR-186 and ZEB1 had a potential binding site (Fig. 3A). Dual luciferase assay showed that the fluorescence intensity in the miR-186 mimic + ZEB1-WT group was significantly reduced compared with the NC mimic + ZEB1-WT group (*p* < 0.01, Fig. 3B), confirming the presence of a binding site between miR-186 and ZEB1. Then, the expression of ZEB1 in miR-186 mimic transfected C666-1 and CNE-2 cells was analyzed, and results showed that compared with the NC mimic group, the protein, and gene expressions of ZEB1 in the miR-186 mimic group were both significantly reduced (*p* < 0.01, Fig. 3C and D), suggesting that miR-186 can target ZEB1 and negatively regulate its expression. In order to further clarify whether ZEB1 is involved in the regulation of NPC by miR-186, the protein and gene expressions of ZEB1 in NPC tissues and cells were also analyzed, and the results showed that the protein and gene expressions of ZEB1 in NPC tissues were significantly increased (*p* < 0.01, Fig. 3E and F). Similarly, ZEB1 protein and gene expression in C666-1 and CNE-2 cells were also significantly increased compared with HNEpC cells (*p* < 0.01, Fig. 3G and H).

### MiR-186 regulated the EMT of NPC cells by targeting ZEB1

**Figure 4:**
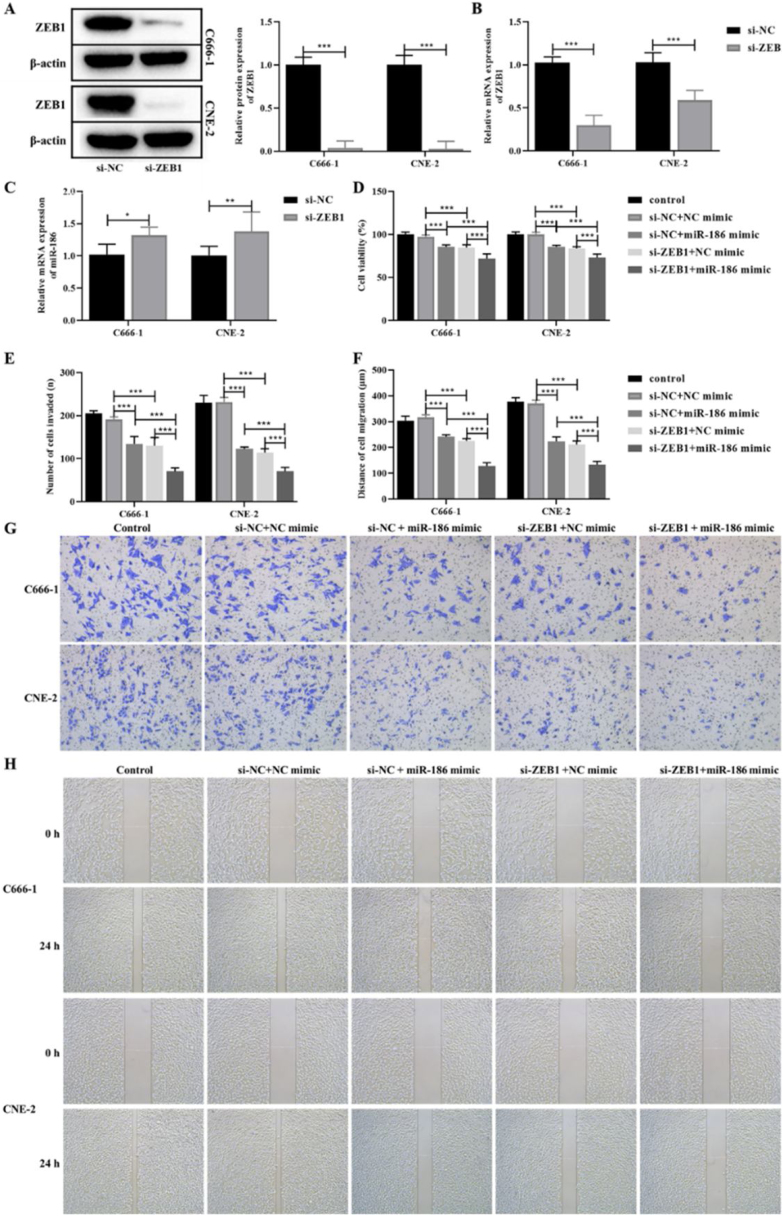
Effects of miR-186/ZEB1 regulatory network on proliferation and metastasis of NPC cells. (A) The protein expression of ZEB1 in NPC cells transfected with si-ZEB1 was detected by WB. Full-length blots/gels are presented in Supplementary Fig. 5. (B) The gene expression of ZEB1 in NPC cells transfected with si-ZEB1 was detected by RT-PCR. (C) The gene expression of miR-186 in NPC cells transfected with si-ZEB1 was detected by RT-PCR. (D) Cell viability was detected by CCK8. (E) The number of cells invaded. (F) Cell migration distance. (G) Representative images of cell invasion measured by transwell. (H) Representative images of cell migration detected by scratch assay. *p < 0.05, **p < 0.01, ***p < 0.001.

**Figure 5:**
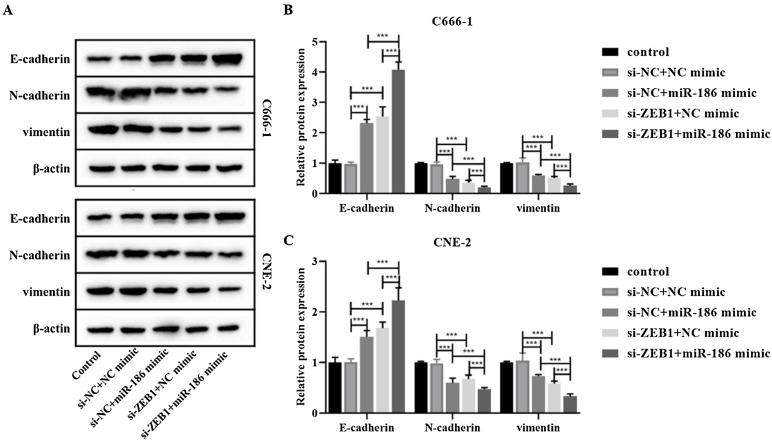
Effects of miR-186/ZEB1 regulatory network on EMT-related protein expression in NPC cells. (A) The protein expressions of E-cadherin, N-cadherin and vimentin were detected by WB. Full-length blots/gels are presented in Supplementary.

The si-RNA targeting ZEB1 was used to intervene the expression of ZEB1 in NPC cells, and the intervention effect was verified by WB and RT-PCR. The results showed that compared with the si-NC group, the protein and gene expressions of ZEB1 in the si-ZEB1 group were both significantly decreased (*p* < 0.001, Fig. 4A and B). And compared with the si-NC group, the expression of miR-186 in the si-ZEB1 group was significantly increased, suggesting that ZEB1 could reverse regulate the expression of miR-186 in NPC (*p* < 0.05, Fig. 4C). The role of the miR-186/ZEB1 regulatory network in NPC cell proliferation and metastasis was further analyzed. CCK8 results showed that miR-186 mimic and si-ZEB1 could significantly inhibit NPC cell viability (*p* < 0.001, Fig. 4D). Transwell and scratch tests showed that miR-186 mimic and si-ZEB1 could significantly inhibit the number of invaded cells and the distance of migration (*p* < 0.001, Fig. 4E‒H). Co-transfected with miR-186 mimic and si-ZEB1 showed more significant inhibitory effects on NPC cells than those of si-NC + miR-186 or si-ZEB1 + NC mimic group (*p* < 0.05, Fig. 4D‒H). In addition, the expressions of EMT-related proteins were analyzed, and results showed that miR-186 mimic and si-ZEB1 could significantly increase the protein expression of E-cadherin and reduce the protein expression of N-cadherin and vimentin in C666-1 and CNE-2 cells (*p* < 0.001, Fig. 5). The regulation effect in the si-ZEB1 + miR-186 mimic group on EMT protein expression was more obvious than that in the si-NC + miR-186 or si-ZEB1 + NC mimic group (*p* < 0.001, Fig. 5).

## Discussion

NPC is a kind of malignant cancer originating from the nasopharyngeal mucosal epithelial, most of which are poorly differentiated or undifferentiated, with high malignancy degree and easy metastasis.^17^ Currently, a lot of studies have shown that the mutation or abnormal expression of miRNA may be linked to cancer and can be used as a key sign for cancer.^18^ The present study found that the expression of miR-186 in NPC tissues and cells was significantly decreased. After transfection with miR-186 mimic, the proliferation activity, invasion, and migration ability, and EMT of NPC cells were significantly inhibited. Further analysis showed that miR-186 could negatively regulate the expression of ZEB1 by targeting its 3′-UTR region and participating in NPC. The miR-186/ZEB1 regulatory network may inhibit the disease process by regulating EMT to inhibit NPC metastasis.

The phenotype of the organisms and the morphological changes of cells, including normal differentiation and the carcinogenesis of cells, are the result of the long-term effects of various factors on the gene regulatory network.^19^, ^20^ The stability of the gene regulatory network plays an important role in the development and maintenance of tissue morphology.^21^ Nearly all cancers have abnormal miRNA–mRNA regulation networks, which may be related to clinical classification, recurrence, and metastasis.^22^, ^23^ The regulatory network of miRNA–mRNA is intimately associated to NPC invasion and metastasis, and aberrant miRNA expressions have been discovered during NPC development.^24^ Cancer suppressor miR-186 is altered in several malignancies. MiR-186 expression varies in cancer type and may be utilized to diagnose and prognosticate cancer.^11^ In addition, studies have confirmed that miR-186 inhibited the metastasis of bladder cancer by targeting NSBP1,^25^ breast cancer by targeting Twist1,^26^ and non-small cell lung cancer by targeting MAP3K2,^27^ playing an important role in the metastasis of multiple cancers. In this work, miR-186 expression in NPC was considerably lowered, inhibiting NPC proliferation, invasion, and migration. In addition, miR-186 directly targets ZEB1 to negatively regulate its expression, and the miR-186-ZEB1 regulatory network is involved in the metastasis of NPC.

ZEB1, a ZEB family member, activates cancer stem cells, regulates apoptosis, promotes angiogenesis, and resists chemotherapy.^28^ ZEB1 is an important transcription factor with zinc finger clusters, which can regulate the transcription of target genes,^29^ and has been confirmed to play a key regulatory role in cancer invasion and metastasis.^30^ ZEB1, which is overexpressed in many malignant tumors, regulates the transcription multiple genes, including vimentin and E-cadherin, to control EMT and accelerate cancer spread.^31^, ^32^ ZEB1 expression is tightly regulated by a variety of pre- and post-transcriptional signaling pathways and molecules.^33^ Current studies have shown that miR-34 could inhibit melanoma by targeting ZEB1,^34^ and miRNA-199b-3p could inhibit ovarian cancer by targeting ZEB1,^35^ suggesting that ZEB1 can be regulated by miRNA to play a key role in cancer. This study confirmed that ZEB1 was also the downstream target gene of miR-186, and miR-186 can target and negatively regulate its expression. As the downstream target gene of miR-186, ZEB1 was significantly high expression in NPC. Intervention with ZEB1 could significantly inhibit the proliferation activity, invasion, and metastasis ability and EMT of NPC.

EMT occurs in most epithelial cell carcinomas during invasion and metastasis.^36^ EMT, which cells lose epithelial properties and gain interstitial ones, contributes to in chronic inflammation, organ fibrosis, cancer invasion, migration, and drug resistance.^37^, ^38^ During the occurrence of EMT, epithelial cells that were originally tightly connected lose their polarity and transform into loosely connected mesenchymal cells with enhanced migration and movement ability, which accelerates the progression of malignant behavior and improves the invasion ability of cancers.^39^ EMT includes the loss of epithelial markers and the increase of interstitial markers. The decreased expression of E-cadherin, and the increased expression of vimentin and N-cadherin are important evidence for the occurrence of EMT.^40^, ^41^ E-cadherin is involved in the maintenance of epithelial tissue integrity and cell polarity, and is widely found in epithelial tissues.^42^ When the expression of E-cadherin is down-regulated, it can cause invasive growth and lead to distal metastasis of cancer cells.^43^ Vimentin is mainly responsible for maintaining the integrity of the cytoskeleton, and is an intermediate filament protein in mesenchymal-derived cells.^44^ The high expression of vimentin indicates the progression of EMT and the occurrence of cancer cell metastasis and invasion.^45^ N-cadherin is involved in biological processes such as cell adhesion, cell signal transduction, cell recognition and cell movement, and its expression level is positively correlated with the degree of metastasis.^46^ This study found that miR-186 could significantly increase the expression of E-cadherin and decrease the expression of vimentin and N-cadherin in C666-1 and CNE-2 cells. In addition, si-ZEB1 further enhanced the inhibitory effect of miR-186, suggesting that miR-186 may inhibit EMT in NPC by inhibiting ZEB1.

## Conclusion

In conclusion, this study found that the expression of miR-186 was significantly low in NPC, and miR-186 mimic could significantly inhibit the proliferation, invasion, and EMT of NPC cells. ZEB1, as a downstream target of miR-186, was significantly highly expressed in NPC. MiR-186 could directly target ZEB1 and negatively regulate its expression, participating in the regulation of NPC cell proliferation and EMT. This study revealed the role of the miR-186/ZEB1 regulatory network in NPC, providing a new target for the diagnosis and treatment of NPC.

## Funding

This work was supported by the Cooperative Scientific Research Funds of Nan Chong City and North Sichuan Medical College (20SXZRKX0006) and Scientific Research and Development Funds of Affiliated Hospital of North Sichuan Medical College (2021ZD013).

## Conflicts of interest

The authors declare no conflicts of interest.

## References

[1] Chen Y.P., Chan A.T.C., Le Q.T., Blanchard P., Sun Y., Ma J. (2019). Nasopharyngeal carcinoma. Lancet (Lond, Engl).

[2] Sung H., Ferlay J., Siegel R.L., Laversanne M., Soerjomataram I., Jemal A. (2021). Global cancer statistics 2020: GLOBOCAN estimates of incidence and mortality worldwide for 36 cancers in 185 countries. CA Cancer J Clin.

[3] Chang E.T., Ye W., Zeng Y.X., Adami H.O. (2021). The evolving epidemiology of nasopharyngeal carcinoma. Cancer Epidemiol Biomarkers Prev.

[4] Guo R., Mao Y.P., Tang L.L., Chen L., Sun Y., Ma J. (2019). The evolution of nasopharyngeal carcinoma staging. Br J Radiol.

[5] Tang L.L., Chen Y.P., Chen C.B., Chen M.Y., Chen N.Y., Chen X.Z. (2021). The Chinese Society of Clinical Oncology (CSCO) clinical guidelines for the diagnosis and treatment of nasopharyngeal carcinoma. Cancer Commun (Lond).

[6] Lee A.W.M., Ng W.T., Chan J.Y.W., Corry J., Mäkitie A., Mendenhall W.M. (2019). Management of locally recurrent nasopharyngeal carcinoma. Cancer Treat Rev.

[7] Toumi N., Ennouri S., Charfeddine I., Daoud J., Khanfir A. (2022). Prognostic factors in metastatic nasopharyngeal carcinoma. Braz J Otorhinolaryngol.

[8] Moreno-Moya J.M., Vilella F., Simón C. (2014). MicroRNA: key gene expression regulators. Fertil Steril.

[9] Saliminejad K., Khorram Khorshid H.R., Soleymani Fard S., Ghaffari S.H. (2019). An overview of microRNAs: biology, functions, therapeutics, and analysis methods. J Cell Physiol.

[10] Ali Syeda Z., Langden S.S.S., Munkhzul C., Lee M., Song S.J. (2020). Regulatory mechanism of MicroRNA expression in cancer. Int J Mol Sci.

[11] Wang Z., Sha H.H., Li H.J. (2019). Functions and mechanisms of miR-186 in human cancer. Biomed Pharmacother.

[12] Lu X., Song X., Hao X., Liu X., Zhang X., Yuan N. (2021). MiR-186-3p attenuates tumorigenesis of cervical cancer by targeting IGF1. World J Surg Oncol.

[13] Guo Z., Lv X., Jia H. (2020). MiR-186 represses progression of renal cell cancer by directly targeting CDK6. Human Cell.

[14] Yao H., Yang Z., Lou Y., Huang J., Yang P., Jiang W. (2021). miR-186 inhibits liver cancer stem cells expansion via targeting PTPN11. Front Oncol.

[15] Wang J., Zhang Y., Ge F. (2021). MiR-186 suppressed growth, migration, and invasion of lung adenocarcinoma cells via targeting dicer1. J Oncol.

[16] Zhang Y., Zhang W. (2020). FOXD1, negatively regulated by miR-186, promotes the proliferation, metastasis and radioresistance of nasopharyngeal carcinoma cells. Cancer Biomark.

[17] Lee H.M., Okuda K.S., González F.E., Patel V. (2019). Current perspectives on nasopharyngeal carcinoma. Adv Exp Med Biol.

[18] He B., Zhao Z., Cai Q., Zhang Y., Zhang P., Shi S. (2020). miRNA-based biomarkers, therapies, and resistance in cancer. Int J Biol Sci.

[19] Nakazawa M.A., Tamada Y., Tanaka Y., Ikeguchi M., Higashihara K., Okuno Y. (2021). Novel cancer subtyping method based on patient-specific gene regulatory network. Sci Rep.

[20] Singh A.J., Ramsey S.A., Filtz T.M., Kioussi C. (2018). Differential gene regulatory networks in development and disease. Cell Mol Life Sci.

[21] Chen Y.R., Huang H.C., Lin C.C. (2019). Regulatory feedback loops bridge the human gene regulatory network and regulate carcinogenesis. Brief Bioinform.

[22] Yousef M., Trinh H.V., Allmer J. (2014). Intersection of MicroRNA and gene regulatory networks and their implication in cancer. Curr Pharm Biotechnol.

[23] Khan S., Jha A., Panda A.C., Dixit A. (2021). Cancer-associated circRNA-miRNA-mRNA regulatory networks: a meta-analysis. Front Mol Biosci.

[24] Liu M., Zhu K., Qian X., Li W. (2016). Identification of miRNA/mRNA-negative regulation pairs in nasopharyngeal carcinoma. Med Sci Monit.

[25] Yao K., He L., Gan Y., Zeng Q., Dai Y., Tan J. (2015). MiR-186 suppresses the growth and metastasis of bladder cancer by targeting NSBP1. Diagn Pathol.

[26] Sun W.J., Zhang Y.N., Xue P. (2019). miR-186 inhibits proliferation, migration, and epithelial-mesenchymal transition in breast cancer cells by targeting Twist1. J Cell Biochem.

[27] Huang T., She K., Peng G., Wang W., Huang J., Li J. (2016). MicroRNA-186 suppresses cell proliferation and metastasis through targeting MAP3K2 in non-small cell lung cancer. Int J Oncol.

[28] Caramel J., Ligier M., Puisieux A. (2018). Pleiotropic roles for ZEB1 in cancer. Cancer Res.

[29] Madany M., Thomas T., Edwards L.A. (2018). The curious case of ZEB1. Discoveries (Craiova).

[30] Cheng L., Zhou M.Y., Gu Y.J., Chen L., Wang Y. (2021). ZEB1: new advances in fibrosis and cancer. Mol Cell Biochem.

[31] Zhang P., Sun Y., Ma L. (2015). ZEB1: at the crossroads of epithelial-mesenchymal transition, metastasis and therapy resistance. Cell Cycle (Georgetown).

[32] Lu J., Fei F., Wu C., Mei J., Xu J., Lu P. (2022). ZEB1: catalyst of immune escape during tumor metastasis. Biomed Pharmacother.

[33] Drápela S., Bouchal J., Jolly M.K., Culig Z., Souček K. (2020). ZEB1: a critical regulator of cell plasticity, DNA damage response, and therapy resistance. Front Mol Biosci.

[34] Xu Y., Guo B., Liu X., Tao K. (2021). miR-34a inhibits melanoma growth by targeting ZEB1. Aging.

[35] Wei L., He Y., Bi S., Li X., Zhang J., Zhang S. (2021). miRNA-199b-3p suppresses growth and progression of ovarian cancer via the CHK1/E-cadherin/EMT signaling pathway by targeting ZEB1. Oncol Rep.

[36] Pastushenko I., Blanpain C. (2019). EMT transition states during tumor progression and metastasis. Trends Cell Biol.

[37] Lu W., Kang Y. (2019). Epithelial-mesenchymal plasticity in cancer progression and metastasis. Dev Cell.

[38] Du B., Shim J.S. (2016). Targeting epithelial-mesenchymal transition (EMT) to overcome drug resistance in cancer. Molecules.

[39] Brabletz S., Schuhwerk H., Brabletz T., Stemmler M.P. (2021). Dynamic EMT: a multi-tool for tumor progression. EMBO J.

[40] Chen T., You Y., Jiang H., Wang Z.Z. (2017). Epithelial-mesenchymal transition (EMT): a biological process in the development, stem cell differentiation, and tumorigenesis. J Cell Physiol.

[41] Bischoff J. (2019). Endothelial-to-mesenchymal transition. Circ Res.

[42] van Roy F., Berx G. (2008). The cell-cell adhesion molecule E-cadherin. Cell Mol Life Sci.

[43] Wong S.H.M., Fang C.M., Chuah L.H., Leong C.O., Ngai S.C. (2018). E-cadherin: its dysregulation in carcinogenesis and clinical implications. Crit Rev Oncol Hematol.

[44] Paulin D., Lilienbaum A., Kardjian S., Agbulut O., Li Z. (2022). Vimentin: regulation and pathogenesis. Biochimie.

[45] Satelli A., Li S. (2011). Vimentin in cancer and its potential as a molecular target for cancer therapy. Cell Mol Life Sci.

[46] Cao Z.Q., Wang Z., Leng P. (2019). Aberrant N-cadherin expression in cancer. Biomed Pharmacother.

