# Supplementary material for: miR-186 regulates epithelial–mesenchymal transformation to promote nasopharyngeal carcinoma metastasis by targeting ZEB1
Source: Braz J Otorhinolaryngol. 2023 Nov 6;90(1):101358. doi: 10.1016/j.bjorl.2023.101358 (PMC10679499; doi:10.1016/j.bjorl.2023.101358)
Supplement: Supplementary file 1 [file mmc1.pdf]

Supplementary Figure 1

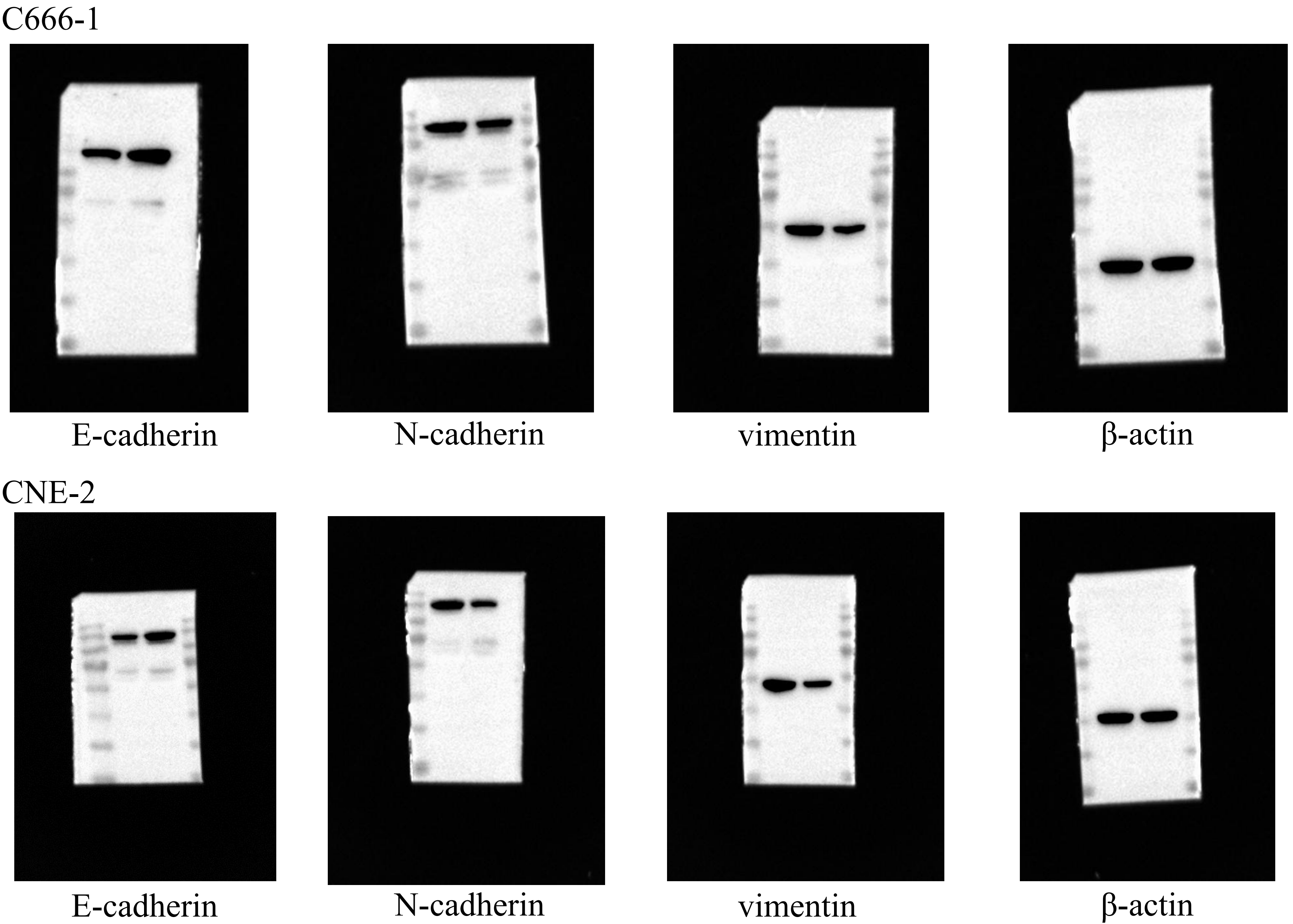

Supplementary Figure 2

C666-1

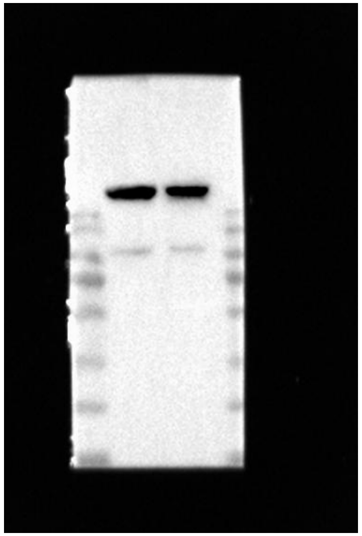

ZEB1

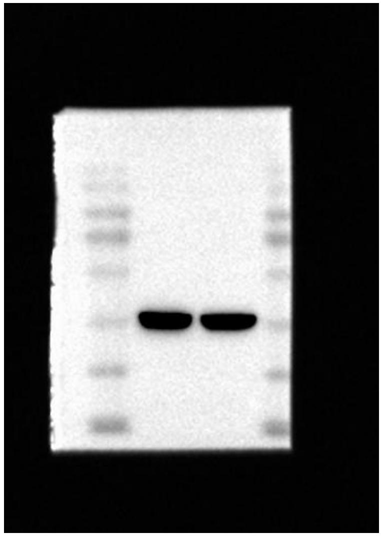

β-actin

CNE-2

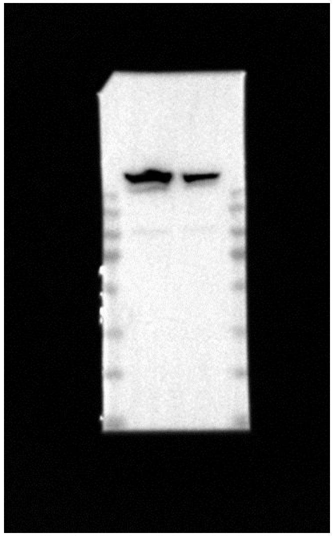

ZEB1

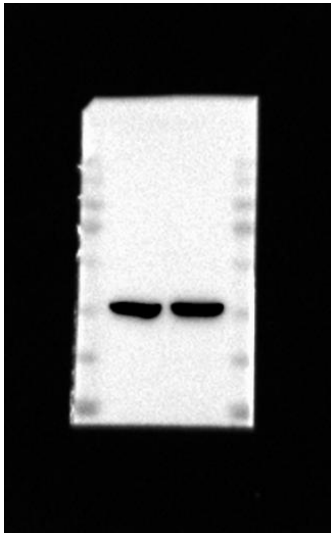

β-actin

Supplementary Figure 3

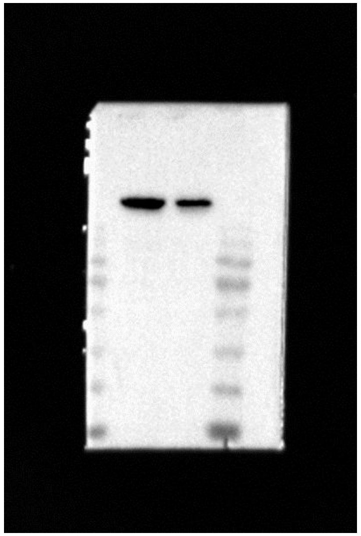

ZEB1

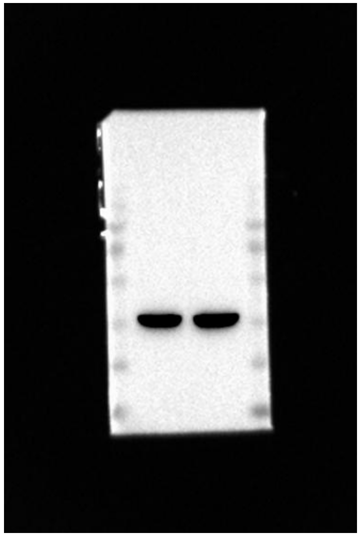

β-actin

Supplementary Figure 4

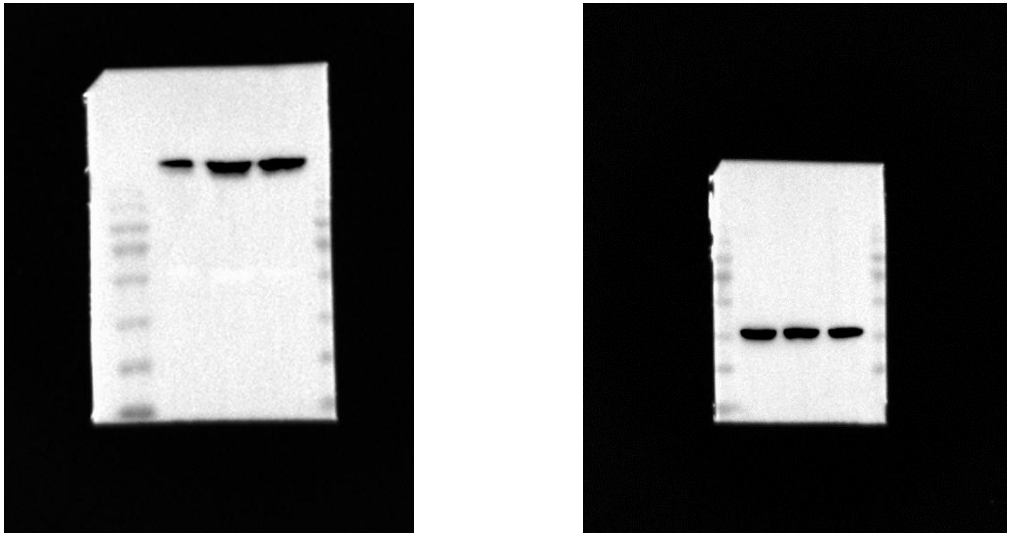

ZEB1 β-actin

Supplementary Figure 5

C666-1

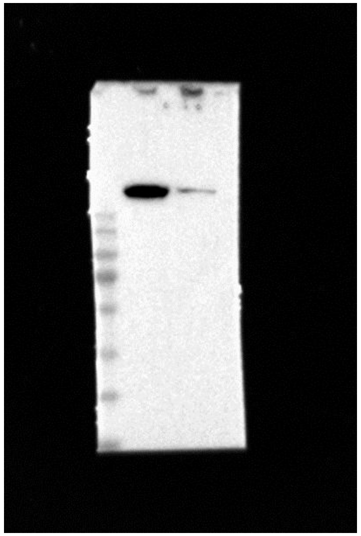

ZEB1

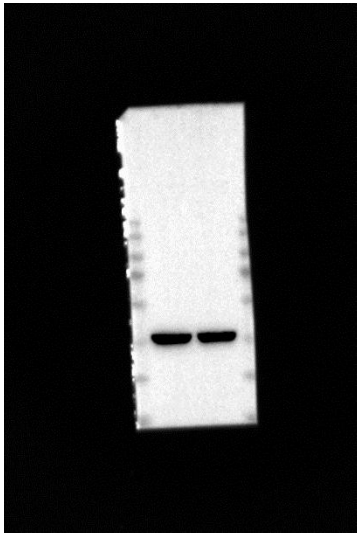

β-actin

CNE-2

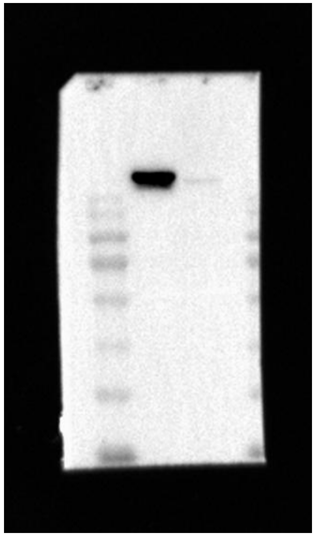

ZEB1

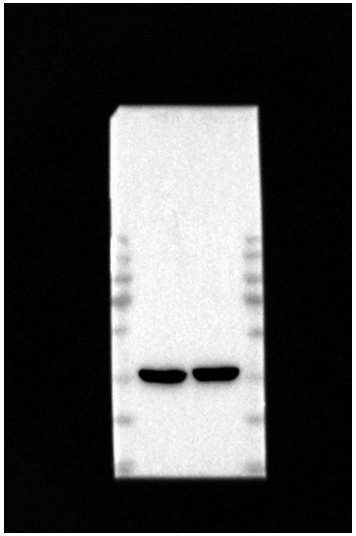

β-actin

Supplementary Figure 6

C666-1

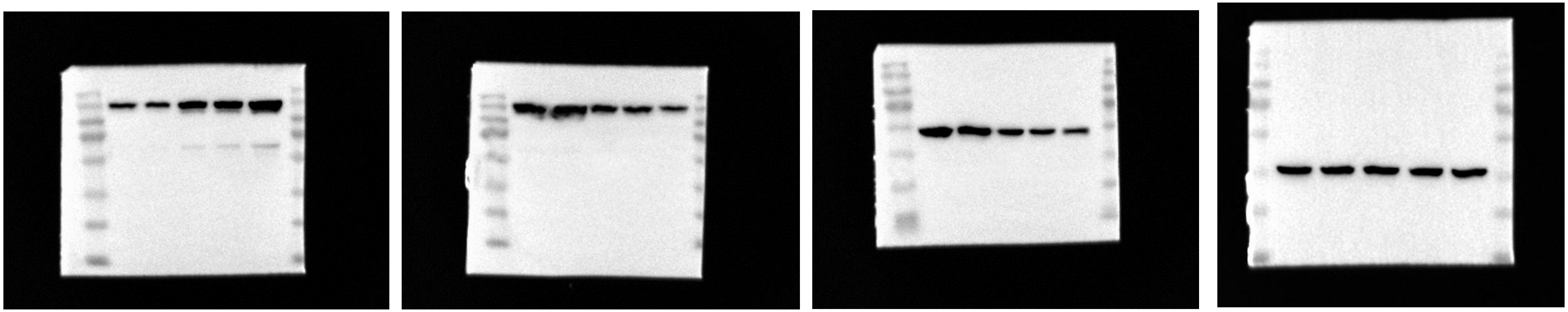

E-cadherin N-cadherin vimentin β-actin

CNE-2

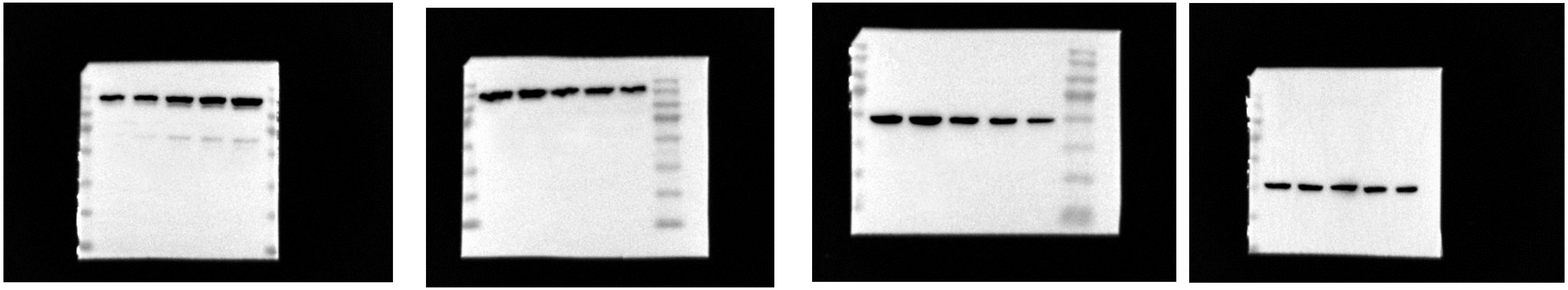

E-cadherin N-cadherin vimentin β-actin
